# In silico prediction of structural changes in human papillomavirus type 16 (HPV16) E6 oncoprotein and its variants

**DOI:** 10.1186/s12860-019-0217-0

**Published:** 2019-08-19

**Authors:** Hugo Alberto Rodríguez-Ruiz, Olga Lilia Garibay-Cerdenares, Berenice Illades-Aguiar, Sarita Montaño, Xiaowei Jiang, Marco Antonio Leyva-Vázquez

**Affiliations:** 1 Molecular Biomedicine Laboratory, Faculty of Chemical-Biological Sciences, Autonomous University of Guerrero, Chilpancingo, Mexico; 2 CONACyT-Autonomous University of Guerrero, Chilpancingo, Mexico; 3 Faculty of Chemical and Biological Sciences, Autonomous University of Sinaloa, Culiacan, Mexico; 4 Division of Birth Cohort Study, Guangzhou Women and Children’s Medical Center, Guangzhou Medical University, Guangzhou, China; 5 Guangzhou-Birmingham Joint Research Center for Birth Cohorts and Disease Cohorts, Guangzhou Medical University, Guangzhou, China; 6 Institute of Cancer and Genomic Sciences, College of Medical and Dental Sciences, University of Birmingham, Birmingham, UK

**Keywords:** HPV16, E6 oncoprotein variants, 3D structure, Molecular modeling, Protein disorder, Molecular dynamics simulations

## Abstract

**Background:**

HPV16 infection is one of the main risk factors involved in the development of cervical cancer, mainly due to the high oncogenic potential of the viral proteins E6 and E7, which are involved in the different processes of malignant transformation. There is a broad spectrum of intratypical variation of E6, which is reflected in its high diversity, biological behavior, global distribution and risk of causing cervical cancer. Experimental studies have shown that the intratypical variants of the protein E6 from the European variants (E-G350, E-A176/G350, E-C188/G350) and Asian-American variants (AAa and AAc), are capable of inducing the differential expression of genes involved in the development of cervical cancer.

**Results:**

An in silico analysis was performed to characterize the molecular effects of these variations using the structure of the HPV16 E6 oncoprotein (PDB: 4XR8; chain H) as a template. In particular, we evaluated the 3D structures of the intratypical variants by structural alignment, ERRAT, Ramachandran plots and prediction of protein disorder, which was further validated by molecular dynamics simulations. Our results, in general, showed no significant changes in the protein 3D structure. However, we observed subtle changes in protein physicochemical features and structural disorder in the N- and C-termini.

**Conclusions:**

Our results showed that mutations in the viral oncogene E6 of six high-risk HPV16 variants are effectively neutral and do not cause significant structural changes except slight variations of structural disorder. As structural disorder is involved in rewiring protein-protein interactions, these results suggest a differential pattern of interaction of E6 with the target protein P53 and possibly different patterns of tumor aggressiveness associated with certain types of variants of the E6 oncoprotein.

**Electronic supplementary material:**

The online version of this article (10.1186/s12860-019-0217-0) contains supplementary material, which is available to authorized users.

## Background

Persistent infection with high-risk human papillomavirus (HR-HPV) is necessary but not sufficient for the development of cervical cancer (CC). High-risk HPV type 16 (HR-HPV16) is the causal agent of more than half of the CC in the world [1]. Its high oncogenic potential is mainly due to the E6 and E7 oncoproteins, as they are key regulators of the cell cycle [2]. Like other HR-HPV, HPV16 has well-preserved distinctive intratypic variants by geographical origin [3], and their global distribution and risk of cervical cancer appear to be dependent on the population [4, 5].

In our group, it has been reported that the intratypical variants of HPV16 E6, namely, E-G350, E-A176/G350, E-C188/G350, AAa and AAc, are the most common and have the most oncogenic potential in the development of CC in southern Mexico, in comparison with the HPV16 E6 reference [6]. Moreover, we analyzed the effects of the expression of HPV16 E6 variants (E-G350, E-A176/G350, E-C188/G350, AAa and AAc) and the E6 reference on global gene expression profiles through an in vitro model, showing that HPV-16 variants are capable of inducing differential expression of host genes involved in the development of CC, such as genes involved in adhesion, angiogenesis, apoptosis, differentiation, cell cycle, proliferation, transcription and protein translation [7].

In protein evolution, a mutation that changes an amino acid is non-synonymous, while a mutation does not change an amino acid is synonymous. Non-synonymous mutations can be detrimental, beneficial or neutral to viral fitness in the host and could often be explained by subtle changes at the protein structural level [8, 9]. In this study, we adopted an in silico approach to evaluate the E6 structural changes. We generated the 3D structures of the five intratypical E6 variants using the crystallized structure of the mutated HPV16 E6 as a template (PDB: 4XR8, chain H from crystal structure of the HPV16 E6/E6AP/p53 ternary complex at 2.25 Å resolution) [10]. We further predicted the structural disorder of the six variants using IUPRED2A [11] and performed molecular dynamics simulations. Our results show that mutations observed in different E6 variants do not significantly alter their 3D structures. However, these non-synonymous mutations slightly modify the structural disorder tendency in the amino- and carboxyl-termini of HPV16 E6, with the amino-terminus being most affected, which is further supported by the molecular dynamics simulation analysis. These changes may lead to differential binding to host P53 proteins and potentially other proteins, which likely affect the oncogenic potential of different HPV16 strains investigated here.

## Results

### Multiple alignment of HPV16 E6 reference and its variants

The protein sequences of each HPV16 E6 variant (reference E6, E-G350, E-A176/G350, E-C188/G350, AAa and AAc) were obtained through a literature search [12]. To compare each variant with the E6 reference, these sequences were subsequently aligned by Clustal W [13].

**Fig. 1:**
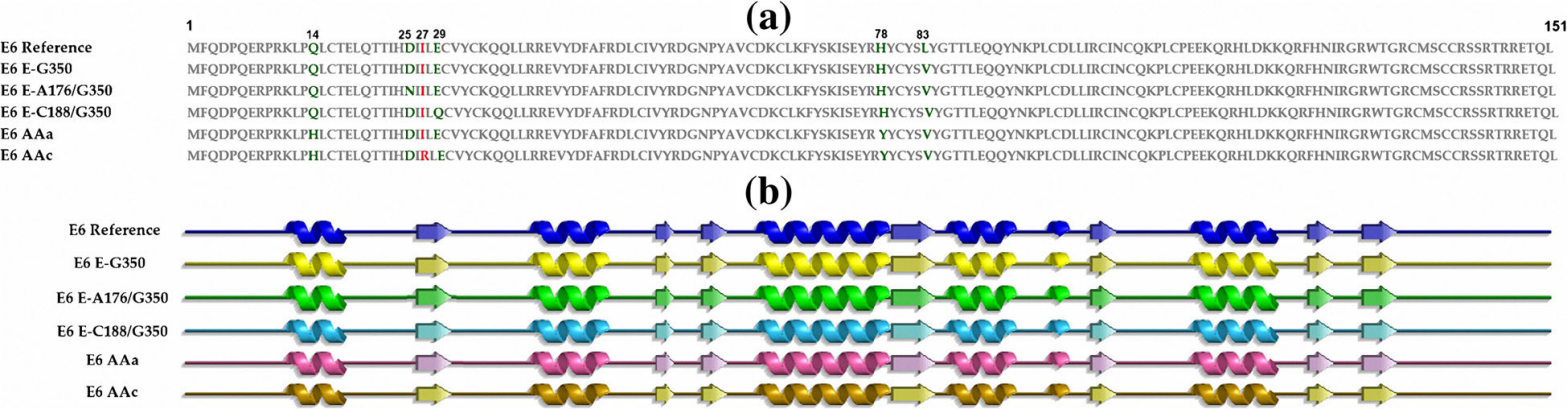
Alignment of the protein sequences and their secondary structures from the reference E6 oncoprotein and its variants.**a** shows the alignment of amino acid sequences of the reference E6 oncoprotein and its intratypical variants. Amino acidic changes of every variant are highlighted in colors. **b** shows the structural alignment of their secondary structures

Figure 1 shows the alignment of protein sequences of all intratypical variants. In Fig. 1a, the primary structures of the E6 reference and its variants are shown. Six non-synonymous sites of amino acid changes are observed among the variants: L83V (E-G350), D25N/L83 V (E-A176/G350), E29Q/L83V (E-C188/G350), Q14H/H78Y/L83V (AAa) and Q14H/I27R/H78Y/L83V (AAc). Although there are amino acid changes in every variant in comparison with the reference, all mutated amino acids remain hydrophilic (green), except the change in I27R (red) located in E6 AAc. This mutation changed isoleucine (I), one of the largest aliphatic amino acids whose structure is often relegated to the hydrophobic core of a protein fold, to a basic amino acid, arginine (R), whose lateral chain is able to have a permanent positive charge in living systems and thus is capable of generating exquisite molecular interactions. In Fig. 1b, the secondary structures of these variants were also aligned, and we can see that their secondary structures are maintained with no changes.

### 3D structures

**Fig. 2:**
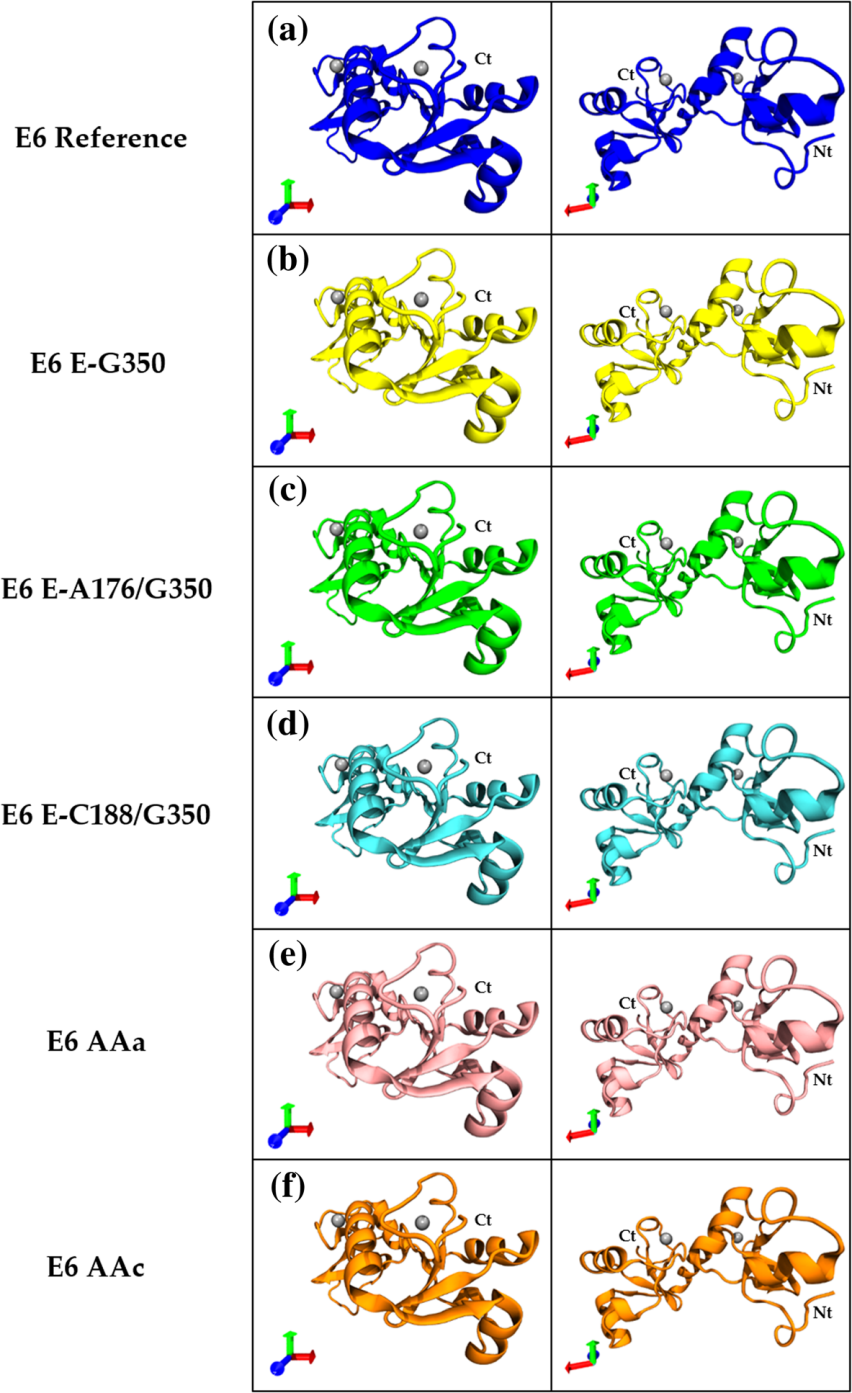
Structures of the reference E6 oncoprotein and five variants**. a** Structure of the E6 reference oncoprotein: blue and its variants: **b** E6-G350: yellow, **c** E6-A176/G350: green, **d** E6-C188/G350: cyan, **e** E6 AAa: pink and **f** E6 AAc: orange. The silver spheres indicate zinc molecules. The orientation of the proteins is indicated by the axes, X: red, Y: green; Z: blue

To understand the structural role of the six non-synonymous mutations, we used the template 3D experimental structure PDB: 4XR8, which contains the 3D structure of a mutated E6 (chain H). To obtain the E6 reference 3D structure, the SCRWL4 program [14] was used to revert all amino acid changes to the E6 reference in the PDB structure. Subsequently, the 3D structures of all variants were obtained in a similar way. In Fig. 2, the structures from the E6 variants and the reference proteins are shown from two different angles: reference (blue), E-G350 (yellow), E-A176/G350 (green), E-C188/G350 (cyan), AAa (pink) and AAc (orange).

**Fig. 3:**
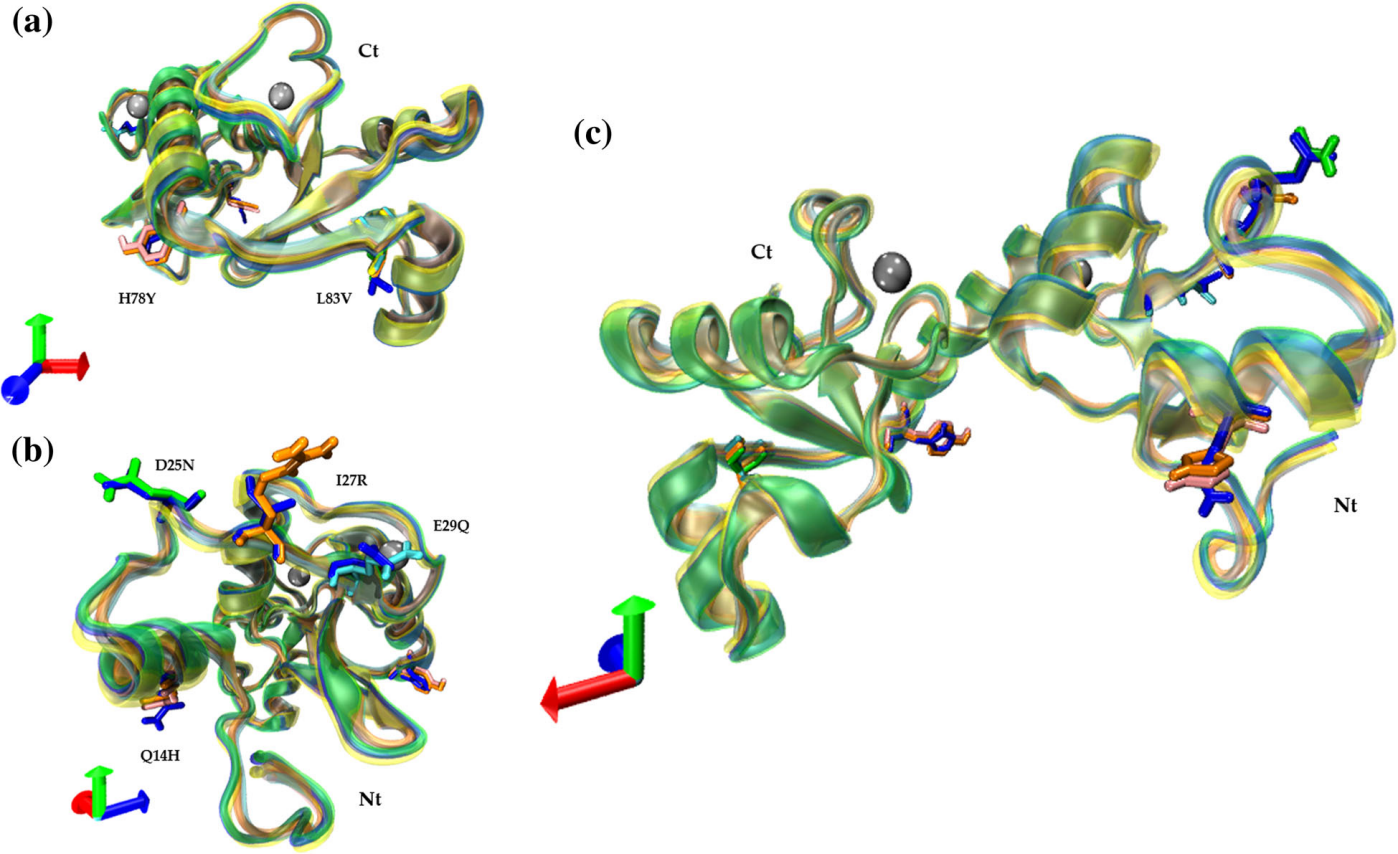
3D Structural alignment of the reference E6 oncoprotein and its variants with amino acid changes highlighted (licorice). **a** Visualization of amino acid changes: H78Y and L83V; **b** Visualization of amino acid changes: Q14H, D25N, I27R and E29Q; **c** Visualization of global overlapping structures. Reference E6 oncoprotein (blue) and its variants: E6-G350 (yellow); E6-A176/G350 (green); E6-C188/G350 (cyan); E6 AAa (pink) and E6 AAc (orange). The silver spheres indicate zinc molecules. The orientation of the proteins is defined by the axes (X: red, Y: green; Z: blue)

In Fig. 3, the 3D structural alignment of each variant to the reference is shown. We highlighted mutated amino acids among the models (licorice). The structural overlap shows different orientations in the axes (X: red, Y: green; and Z: blue). Two amino acid mutations, H78Y (AAa and AAc) and L83V (all variants), are shown in Fig. 3a. Additionally, it is possible to identify changes in the amino acids Q14H (AAa and AAc), D25N (E-A176/G350), I27R (AAc) and E29Q (E-C188/G350) in Fig. 3b. Finally, in Fig. 3c, we structurally aligned all six protein structures from the E6 reference and all five variants (E-G350, E-A176/G350, E-C188/G350, AAa and AAc). Interestingly, all models have the same general structural conformation, and the non-synonymous mutations of the variants do not have a visible effect on the 3D structure of E6, which clearly demonstrates that the 3D structure of the protein seems to be preserved despite variations in amino acids.

**Fig. 4:**
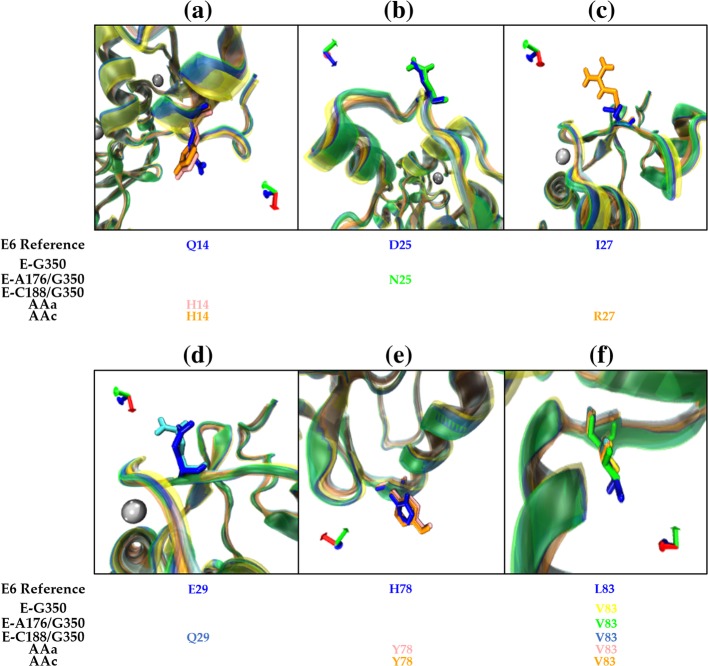
Amino acids from the non-synonymous sites among the E6 reference and its variants**. a** Two different amino acids at position 14 (Q14) in the sequence alignment between the reference E6 oncoprotein and AAa and AAc variants; **b** Two different amino acids at position 25 (D25) between the reference E6 oncoprotein and alternative E6-A-176/G350 are shown; **c** Amino acid at position 27 (I27) of the E6 oncoprotein reference differs in the variant AAc by R27; **d** The amino acid at position 29 (E29) differs in the variant E6-C188/G350; **e** shows the amino acid at position 78 (H78) that differs in the AAa and AAc variants; **f** shows the amino acid at position 83 (L83) which is mutated in all variants. The same colors are used across panels for different amino acids from the same variant. Different colors are used as follow: E6 reference oncoprotein (blue), E6-G350 (yellow), E6-A176/G350 (green), E6-C188/G350 (cyan), E6 AAa (pink) and E6 AAc (orange). The silver spheres indicate zinc molecules. The orientation of the amino acids is defined by the axes shown (X: red, Y: green; Z: blue)

In Fig. 4, from the above aligned protein structures (Fig. 3), we isolated the aligned amino acid changes in every variant in comparison with the E6 reference. The subtle modifications in the orientation of the side chains are due to a particular amino acid change whose functional groups belong to the same biochemical group; however, a drastic change occurs in the lateral chain in the change of I27R in E6 AAc due to amino acids of different biochemical behavior.

### 3D structure evaluation

**Fig. 5:**
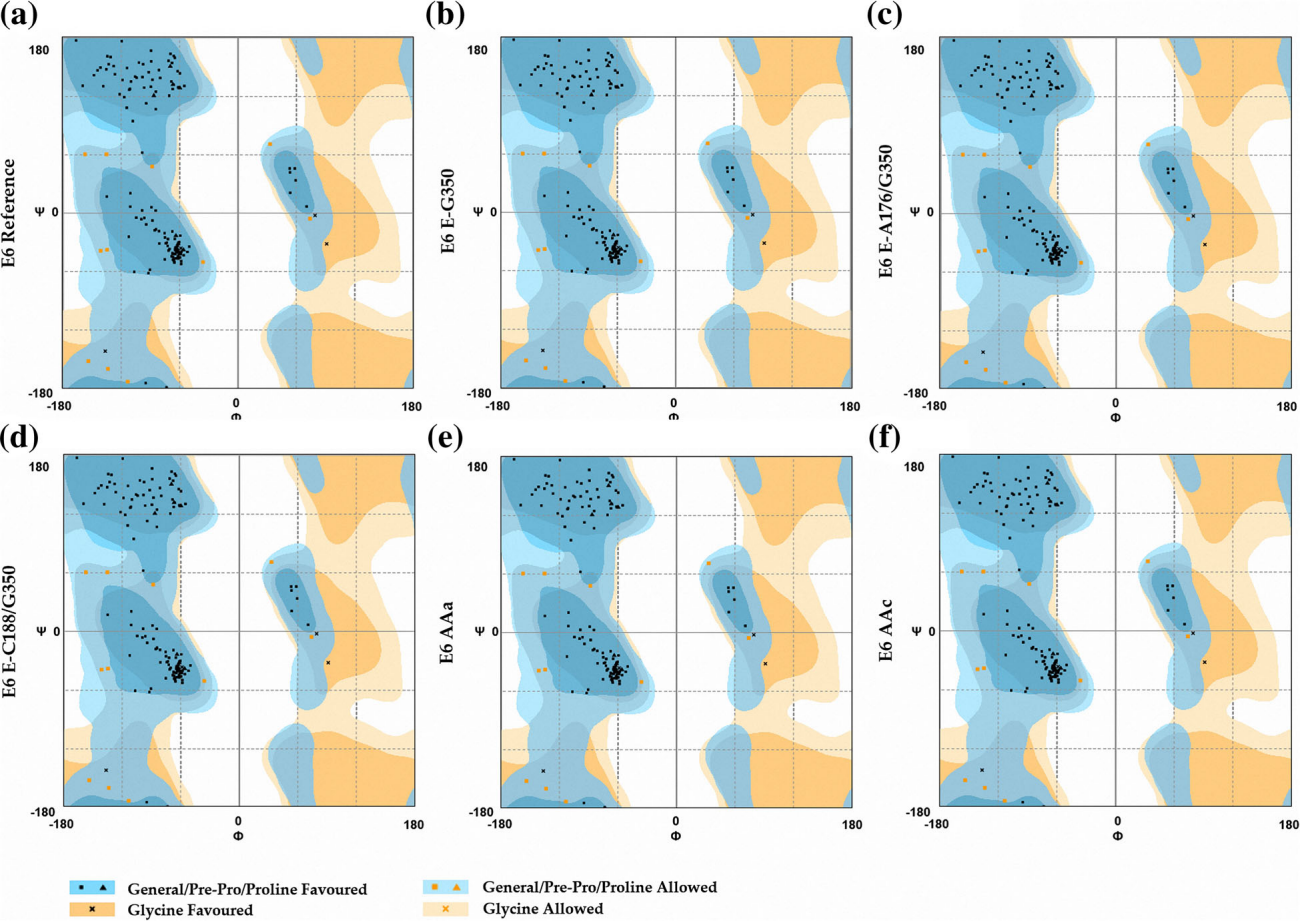
Ramachandran plot analysis. The analysis showed 92.6% of amino acids in all variations were located in the region highly favored, 7.4% in the favored region and no atypical amino acid in the not favored region. **a** E6 Reference; **b** E6-G350; **c** E6-A176/G350; **d** E6-C188/G350; **e** E6 AAa and **f** E6 AAc

**Fig. 6:**
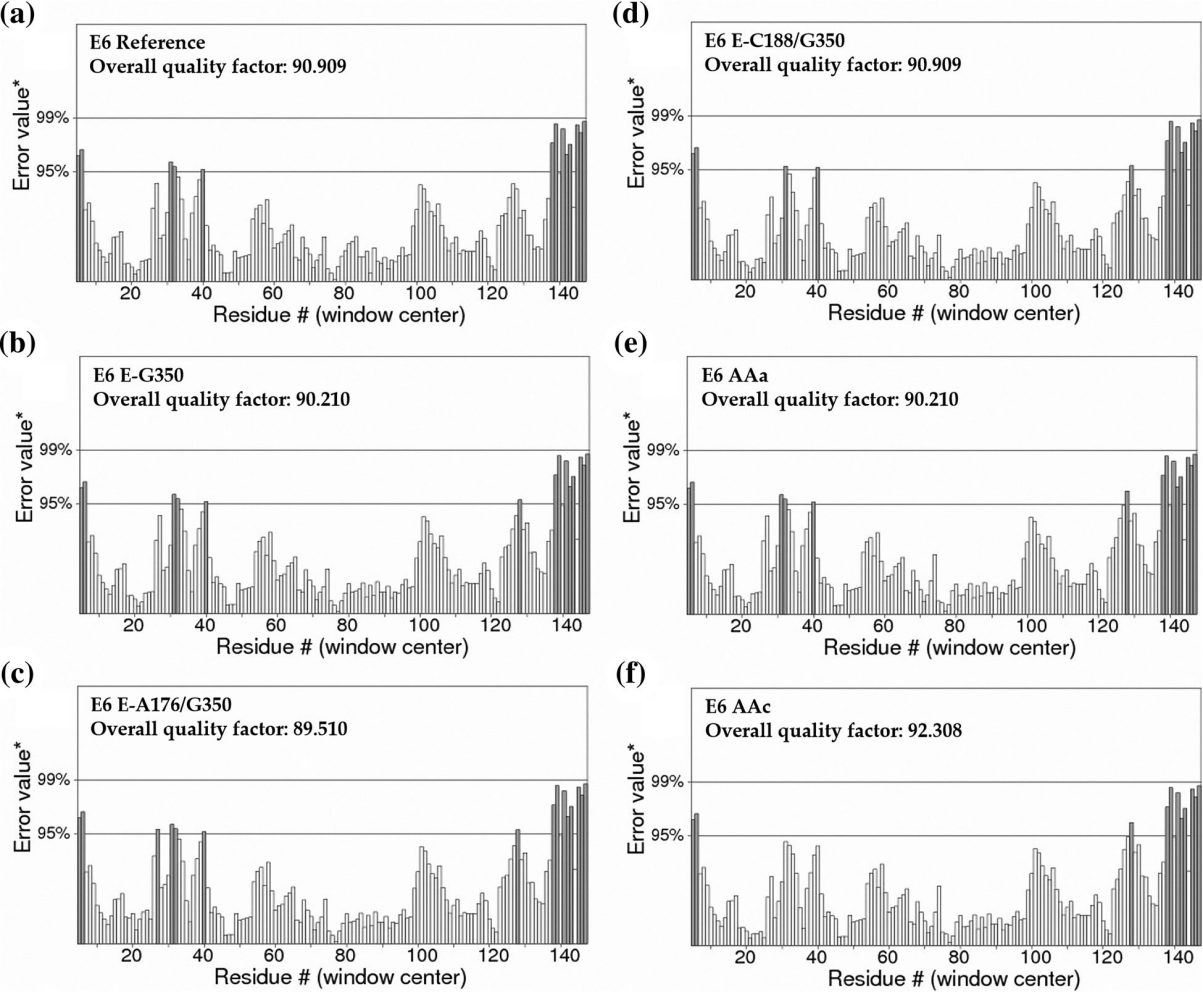
ERRAT server analysis. Graphics show the overall quality factor of the reference E6 oncoprotein and its variants. **a** E6 oncoprotein reference; **b** E6-G350; **c** E6-A176/G350; **d** E6-C188/G350; **e** E6 AAa and **f** E6 AAc. *On the error axis, two lines are drawn to indicate the confidence with which it is possible to reject regions that exceed that error value. Overall quality factor, is expressed as the percentage of the protein for which the calculated error value falls below the 95% rejection limit

To evaluate and validate the accuracy of the predicted 3D structures of the HPV16 E6 reference and its variants, Rampage and ERRAT servers were used. Ramachandran plot analysis of the HPV16 reference and its variants are also shown. In Fig. 5, the disallowed regions are colored pale-yellow. Red color indicates low-energy regions. Brown color indicates allowed regions, and yellow indicates the so-called generously allowed regions. This analysis showed 92.6% amino acids in the highly favored region, 7.4% in the favored region and no atypical amino acids in the unfavored region, which suggest that the generated models are reliable. Evaluation of the quality of the models generated using the ERRAT server showed an overall quality factor that was different for each one of the variants (Fig. 6), with a value greater than 50 considered favorable to the E6 reference. E-C188/G350 presented a quality factor of 90.909, while for E6 E-G350, the quality factor was 90.210; however, E6 E-A176/G350 presented the lowest value of quality factor of all variants at 89.510. Finally, for the variants AAa and AAc, the quality factors were 90.210 and 92.308, respectively. Despite the fact that all the variants obtained a favorable value, the variant AAc obtained the highest overall quality factor.

### Physical and chemical properties

To analyze the possible modifications in the physicochemical properties of the HPV16 E6 reference and its variants, we submitted the primary sequences to the Expasy’s ProtParam Tool server. This server allows the theoretical prediction of several physical and chemical parameters from a given primary protein sequence, such as molecular weight (MW), theoretical isoelectric point (Ip), amino acid composition, atomic composition, extinction coefficient (EC), estimated half-life, instability index (II), aliphatic index (AI) and grand average of hydropathicity (GRAVY).

As a result, the theoretical value of the isoelectric point (pI) calculated for the reference HPV16 E6 reference as well as the different variants were in the range of 9.01 to 9.10, indicating that the E6 reference and variants have a basic isoelectric point. The EC indicates how much light a protein absorbs at a certain wavelength but is also a value used to determine the concentrations of proteins in solution to perform processes of purification. This property was different between the variants, introducing an EC value of 21,275 M^− 1^ cm^− 1^ at 280 nm for the E6 reference and the variants E-G350, E-A176/G350, E-C188/G350, and an EC value of 22,765 M^− 1^ cm^− 1^ at 280 nm for the variants AAa and AAc. The II provides an estimate of the stability of a protein in a test tube; a protein whose II is smaller than 40 is predicted to be stable, while a value above 40 predicts a highly unstable protein. All variants analyzed, including the reference, presented a high instability index: E6 reference: 73.25; E-G350: 72.75; E-A176/G350: 77.23; and E-C188/G350: 71.50. However, the AAa and AAc variants showed the lowest rates of instability at 67.61 and 66.34, respectively. The AI, which is defined as the relative volume occupied by the aliphatic side chains (alanine, valine, leucine, and isoleucine), is considered a positive factor for the increase in the heat stability of globular proteins. The AI value of the E6 reference was the largest at 70.99, followed by the AI of the variants E-G350, E-A176/G350, E-C188/G350 and AAa, which was 70.33. Finally, the AI of the AAc variant was the lowest, with a value of 67.75. The GRAVY value for a peptide or protein is the sum of the hydropathic values of all the amino acids divided by the number of residues in the sequence, indicating the feature hydrophobic (positive values) or hydrophilic (negative values) of a protein while taking into account the length of the sequence of amino acids. This value for the proteins analyzed was − 0.734 for the reference, − 0.732 for the variants E-G350, E-A176/G350, E-C188/G350, − 0.717 for variant AAa and − 0.777 for variant AAc, with the latter variant being the most hydrophilic.

### Structural disorder prediction of E6 reference and variants

**Fig. 7:**
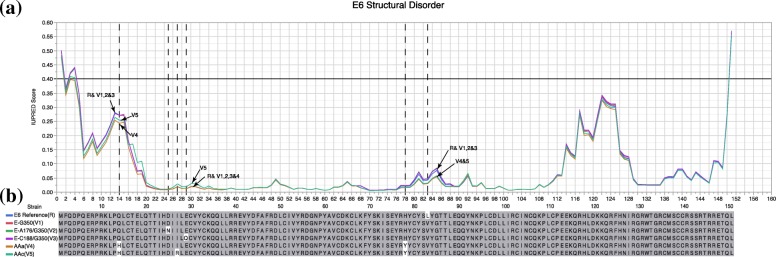
Protein disorder analysis**. a** Graphic shows the disorder tendency for each amino acid predicted by UPRED2A of the E6 reference and its variants E6-G350; E6-A176/G350; E6-C188/G350; E6 AAa and E6 AAc. **b** The primary sequences of E6 reference and its variants are showed, the white boxes highlighting the amino acid changes on each variant in comparison with E6 reference

Proteins are known to have different levels of structural disorder either for the whole protein or within their protein domains. There are specific amino acids contributing to structural disorder at different levels, which can lead to differential interactions with target proteins [15]. Here, we investigated the structural disorder in the E6 reference and its variants using IUPRED2A [11]. Figure 7a shows that the structural disorder for each variant does not deviate significantly from each other. However, there are several noticeable differences due to non-synonymous mutations. Around site 14, the E6 reference shares similar disorder scores with variants E-G350, E-A176/G350 and E-C188/G350, which have higher disorder tendencies than variants AAa and AAc. AAc has a higher disorder tendency than variant AAa. Around site 29, variant AAc has the highest disorder tendency while the rest has the same disorder tendency. From sites 78 to 90, the reference has similar disorder tendencies as variants E-G350, E-A176/G350 and E-C188/G350, which have higher disorder scores than AAa and AAc variants. Even when the prediction analysis of IUPRED2A showed no significant variations related to protein disorder, it is noticeable that all variations are located in the regions where amino acids are modified, and most disorder prediction sites could explain the subtle structural changes that could affect the interaction of E6 with host proteins (Fig. 7b).

### Molecular dynamics simulation analysis

**Fig. 8:**
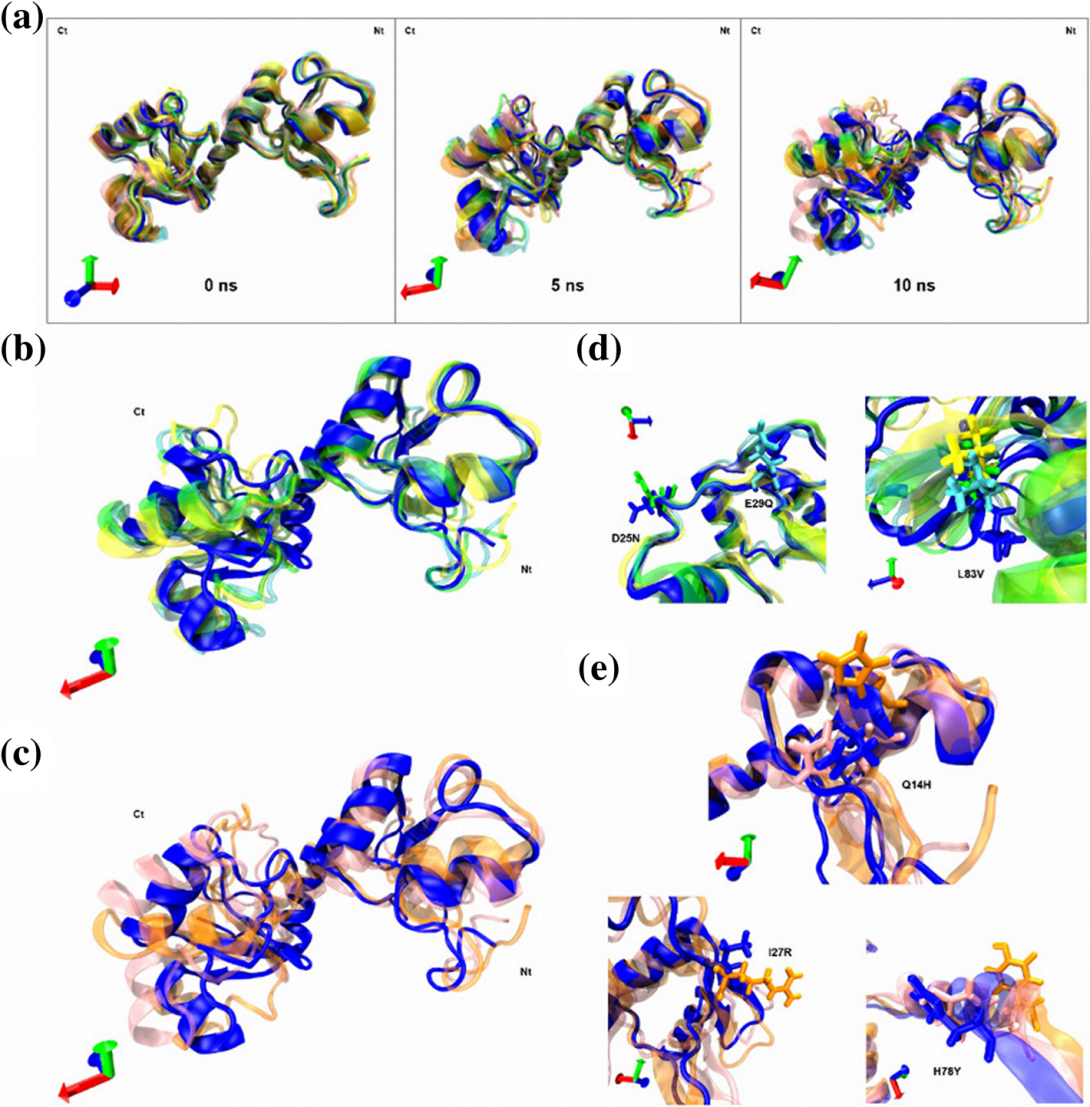
Molecular dynamics simulations of E6 oncoproteins. **a** Shows a structural overlap of all variants at 0, 5 and 10 ns. **b**, **c** The structural overlap of reference E6 oncoprotein with EUR variants and AA variants are shown. **d** Visualization of amino acids conformational changes: D25N, E29Q and L83V. **e** Visualization of amino acids conformational changes: Q14H, I27R and H78Y. The same colors of Fig. 2 are used for each variant

As the SCRWL4 program only optimizes sidechain conformations when building homology models, to further understand the effect of the six non-synonymous mutations of E6 oncoprotein on side chain and backbone conformations, MD simulations of 10 ns of the reference E6 oncoprotein and variants E-G350, E-A176/G350, E-C188/G350, AAa and AAc were performed to analyze the structural impact of the intratypical variations, and the structural alignments are shown (Fig. 8). The frames of 0, 5 and 10 ns were obtained to visualize the perturbations on backbones (Fig. 8a-c) and side chains (Fig. 8d-e) during the simulations, where most variations are located in the side chains of the non-synonymous mutations (Fig. 8d-e). The Fig. 8a shows certain instability in amino- and carboxyl-termini after structurally aligning all variants. Particularly, there is a notable increase of flexibility (disorder) in amino- and carboxyl-termini of all variants through the simulation consistent with the disorder prediction, being more visible in the Asian American variants (Fig. 8c) when compared with the European variants (EUR; E-G350, E-A176/G350, E-C188/G350) (Fig. 8b). Moreover, the most representative side chain variations are shown in Fig. 8d for EUR variants (D25N, E29Q and L83V), and in Fig. 8e for AA variants (Q14H, I27R and H78Y).

## Discussion

High-risk mucosal HPV infections are responsible for the majority of cervical, anal, rectal, and penile cancers, as well as an increasingly high proportion of oropharyngeal cancers. The two main viral HPV oncogenes required to establish and maintain the tumorigenic phenotype encode two early expressed oncoproteins, called E6 and E7 [16]. It is known that the HPV16 E6 reference is involved in several biological processes of malignant transformation, such as cell cycle, apoptosis, DNA repair, immune response, organization of chromatin, and cell communication [17, 18].

Our initial structural analysis based on homology modeling did not find any significant change of the protein structures of the variant E6 proteins. We subsequently used Expasy’s ProtParam Tool server to predict the physicochemical properties of these proteins. It was possible to predict some physical and chemical parameters from the E6 reference and their variants, which showed minimal differences in the isoelectric point, but when the high index of instability was analyzed, AA variants (a and c) showed the highest values of instability. The instability index could be used as an indirect measure of the in vivo half-life of a protein [19]. Proteins that have an in vivo half-life of less than 5 h have been shown to have an instability index of more than 40, whereas those that have an in vivo half-life of more than 16 h have an instability index of less than 40 [20]. The values obtained for the proteins in this work are in the range of 66–69, and these theoretical predictions suggest a long half-life at an average of 5 h for variants of the E6 oncoprotein. Finally, the hydrophobicity was determined using the GRAVY index (the sum of hydropathic values of all amino acids divided by the protein length), whose values could be related to the solubility and aggregation of a protein, which can greatly affect the recovery efficiency in the recovery process [21, 22]. In all E6 analyzed, the negative values are associated with their hydrophilic behavior and could be related to the cytoplasmic localization or localization in the lumen of certain organelles.

In the UniProt database there are 9 PDB structures corresponding to HPV16 E6 (1VZN, 2FK4, 2KPL, 2LJX, 2LJY, 2LJZ, 4GIZ, 4JOP and 4XR8), where we used 4XR8 [10]. Unlike the rest of the crystallized structures, which correspond to the amino or carboxyl ends, or to binding domains, the 4XR8 structure corresponds to the structure of a quaternary complex HVP16 E6 / E6AP / p53, in which the domains associated with the interaction with E6AP and P53 are shown. This is the only complex allows us to get the complete structure of E6 with their respective interactions with P53 and perform homology modeling, which further supported our analysis of the molecular dynamics simulations. Our study reveals that these non-synonymous mutations on E6 from these highly oncogenic variants cause subtle structural and potential functional changes, which may play an important role in mediating how they interact with P53. These differential interactions may subsequently determine the level of susceptibility of developing cervical cancer or other related cancers. Particularly, it can be clearly seen that Q14H mutation is right at the binding interface of E6-P53core, which is located at the sub-interfaces I of the binding interface (Additional file 1: Figure S1) [10]. Previous functional studies demonstrate this site (Q14A) plays an important role in P53 binding and subsequent degradation [10]. Other mutations located around this and other active sites may allosterically contribute to P53 binding and degradation [10, 23]. Further docking analysis shows that although the site of interaction with E6AP-E6 does not change, the interaction pattern between E6-E6AP and P53 does change, potentially due to the subtle structural effect of these pathogenic mutations at both the N- and C-termini of the viral E6 protein (unpublished data). However, we caution that due to limited number of E6 structures currently available, our homology model may not be entirely accurate. This can be improved in the future when more full-length E6 structures are available.

There are proteins that have 3D structural conformation highly heterogeneous and are known to have different levels of structural disorder, from slightly flexible to intrinsically disordered [24]. Their structural conformations depend on different conditions triggered by pH, temperature, redox potential, mechanical force, light exposure and various types of interactions [25]. The biological function of these proteins is directly linked to their foldability during interaction with their targets. HPV-16 E6 is a protein with regions of different structural disorder levels, particularly the N- and C-termini as evidenced by the molecular dynamics simulations. In this in silico analysis, the amino acid changes of variants are predominantly located in the N-terminal region, we suggest that a subtle change in structural disorder tendency could be enough to confer variations in HPV16’s oncogenic potential, which should aid future functional studies of these types of mutations.

The present study provides a framework to evaluate the mechanism by which HPV16 E6 structural disorder could be related to differential molecular interactions to key host tumour suppressive and/or oncogenic pathways and, most importantly, to establish a new point of view about the design of new therapeutic strategies based on viral oncoproteins with plasticity as molecular targets.

## Conclusions

In silico analysis shows that the primary sequences of all HPV E6 variants analyzed have variations in the first 83 amino acids; however, their general 3D structure does not change. The molecular modeling and molecular dynamics simulations of these protein variants showed minimal changes in general structure, but broad changes in their physicochemical parameters, which are possibly involved in the differential pattern of interactions with protein targets.

## Methods

### Obtaining the target sequence and its variants

The primary sequence of amino acids in the HPV16 E6 reference was obtained from the UniProt database (http://www.uniprot.org/) with accession number P03126. The E6 oncoprotein consists of 477 nucleotides that encode 151 amino acids (aa), presents two zinc fingers and has a PDZ domain in its carboxyl-terminus [26]. The changes in amino acids in each variant analyzed are shown in Table 1. The amino acid sequences of the E6 variants E-G350, E-A176/G350, E-C188/G350, AAa and AAc were reported by Huertas-Salgado et al. [12]. To submit the sequences to alignment, an amino acid substitution from the reference sequence of HPV16 E6 was performed.

**Table 1:** Amino acids differentials between the sequence of the E6 reference and variants of HPV16

E6 Reference 151 aa	14	25	27	29	78	83
	Q	D	I	E	H	L
E-G350						V
E-A176/G350		N				V
E-C188/G350				Q		V
AAa	H				Y	V
AAc	H		R		Y	V

### Generation of 3D structures

The complete HPV16 E6 oncoprotein three-dimensional (3D) structure is found in the Protein Data Bank (PDB) with the access code 4XR8, chain H (http://www.rcsb.org/pdb/) [10]. To obtain the secondary structure, the primary sequence (P03126) and its respective variants were submitted to PDBsum server [27]. The sequence of the E6 oncoprotein in this PDB is called E6 4C/4S and has four point mutations (Ser80Cys, Ser97Cys, Ser111Cys, and Ser140Cys) [10]. To analyze the mutations of the E6 oncoprotein variants, E-G350, E-A176/G350, E-C188/G350, AAa, and AAc, and the E6 reference, the SCWRL4 program was used [14].

### Validation of modeled structures

The accuracy of the predicted models was evaluated by Ramachandran plot using the RAMPAGE server (http://mordred.bioc.cam.ac.uk/~rapper/rampage.php) [28] and ERRAT server (http://services.mbi.ucla.edu/ERRAT/) [29] to check the quality of these models.

#### Calculation of physiochemical properties

To calculate the physical and chemical parameters of proteins, such as molecular weight, theoretical isoelectric point, amino acid composition, atomic composition, extinction coefficient, estimated half-life, instability index, aliphatic index and grand average of hydropathicity, we used the Expasy’s ProtParam Tool server (https://web.expasy.org/protparam/) [30].

#### Visualization

All 3D structures were visualized in the VMD 1.9.1 program (Visual Molecular Dynamics), which is a molecular visualization program for displaying, animating, and analyzing large biomolecular systems using 3D graphics and built-in scripting (http://www.ks.uiuc.edu/Research/vmd/) [31].

### Structural disorder prediction

The IUPRED2A is based on the calculation of the pairwise amino acid interaction energies in a given length. So, a single amino acid change (e.g., Q14H mutation) could potentially change the disorder tendency in that region [32]. This server takes amino acid sequence in FASTA format as input. The results are returned in either text or graphical format, specifying the disorder tendency of each residue along the sequence. This score can take a value between 0 and 1. Residues with a predicted score above 0.4 are considered disordered [33].

### Molecular dynamics simulation

We used the NAMD 2.8 program [34] to perform the molecular dynamics (MD) simulations of the reference E6 oncoprotein and variants E-G350, E-A176/G350, E-C188/G350, AAa and AAc**.** We collaborated with the Laboratory of Molecular Modeling and Bioinformatics of the Facultad de Ciencias Químico Biológicas de la Universidad Autónoma de Sinaloa, using the Hybrid Cluster Xiuhcoatl (http://clusterhibrido.cinvestav.mx) LANCAD and GPU-CUDA with video cards graphics NVIDIA Tesla C2070/Tesla C2075. The force fields CHARMM22 and CHARMM27 [35] were used for topologies and lipids. The TIP3 model was used for water molecules. The system was solvated using the psfgen plugin in the VMD program [31]. To add water molecules and ions to neutralize the system, we added 9725 water molecules and 15 Cl^−^ for the E6 reference. For variant E-G350, we added 7924 water molecules and 15 Cl^−^. For variant E-A176/G350, we added 7924 water molecules and 16 Cl^−^. For variant E-C188/G350, we added 7926 water molecules and 15 Cl^−^. Finally, for variant AAa we added 7924 water molecules and 15 Cl^−^. For variant AAc, we added 7860 water molecules and 12 Cl^−^. Moreover, for all structures two Zn^2+^ were added. The system was submitted to minimization energy for 1000 steps followed by equilibration for 1 ns under constant temperature and pressure (NPT) with protein and lipid atoms restrained. Molecular dynamics simulations were run for 10 ns using the NTV ensemble, considering E6 and its variants as soluble proteins. The frames from 0, 5 and 10 ns were obtained with the Carma software [36]. The 3D structures visualization and structural alignment were performed by VMD software [31].

## Additional file

Additional file 1: **Figure S1.** Illustration of the non-synonymous mutations on E6 structure (colored grey) in complex with the p53core (colored wheat). Mutations Q14H, D25N, I27R and E29Q are colored red for the N-terminus mutations, while mutations H78Y and L83 V are colored green for C-terminus mutations. (A) the side view of the six non-synonymous mutations. (B) top view of the six non-synonymous mutations. PDB 4XR8 and PyMOL 2.3.0 were used to map these mutations. (PDF 2443 kb)

## Data Availability

Not applicable.

## Abbreviations

3D: Three Dimensional
CC: Cervical Cancer
HPV16: Human Papillomavirus Type 16
HR-HPV: High Risk Human Papillomavirus
PDB: Protein Data Bank
