# Supplementary figures and images for: In silico prediction of structural changes in human papillomavirus type 16 (HPV16) E6 oncoprotein and its variants
Source: BMC Mol Cell Biol. 2019 Aug 19;20:35. doi: 10.1186/s12860-019-0217-0 (PMC6700771; doi:10.1186/s12860-019-0217-0)

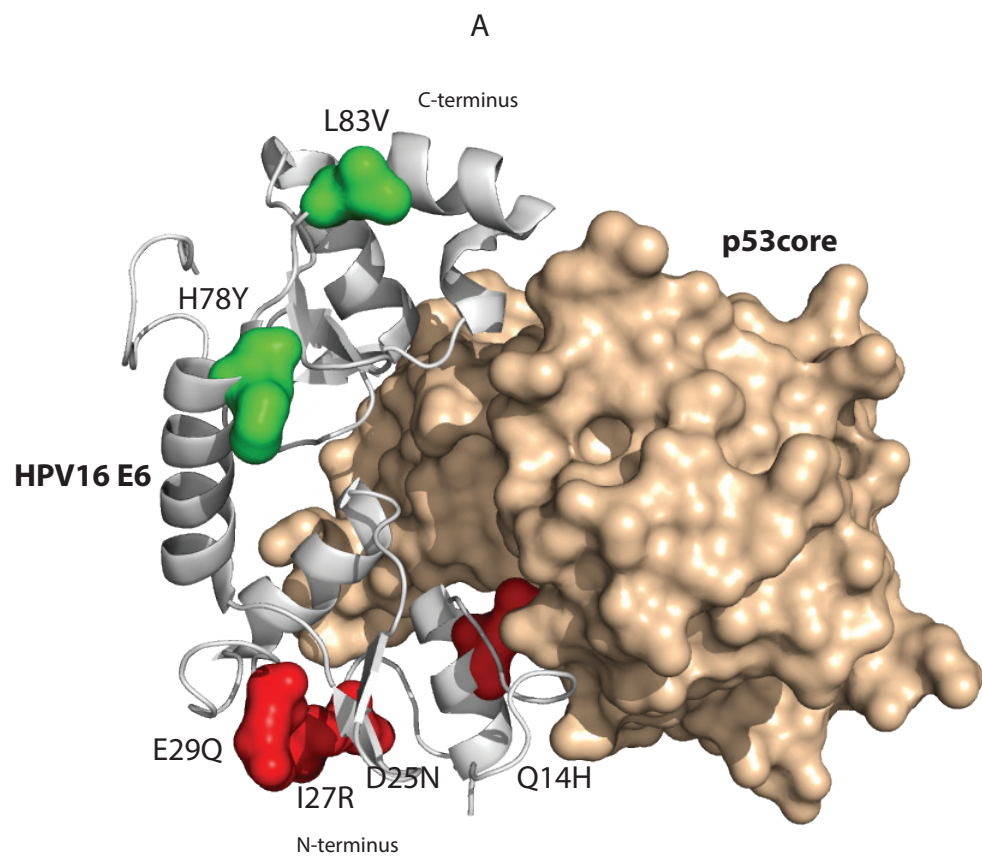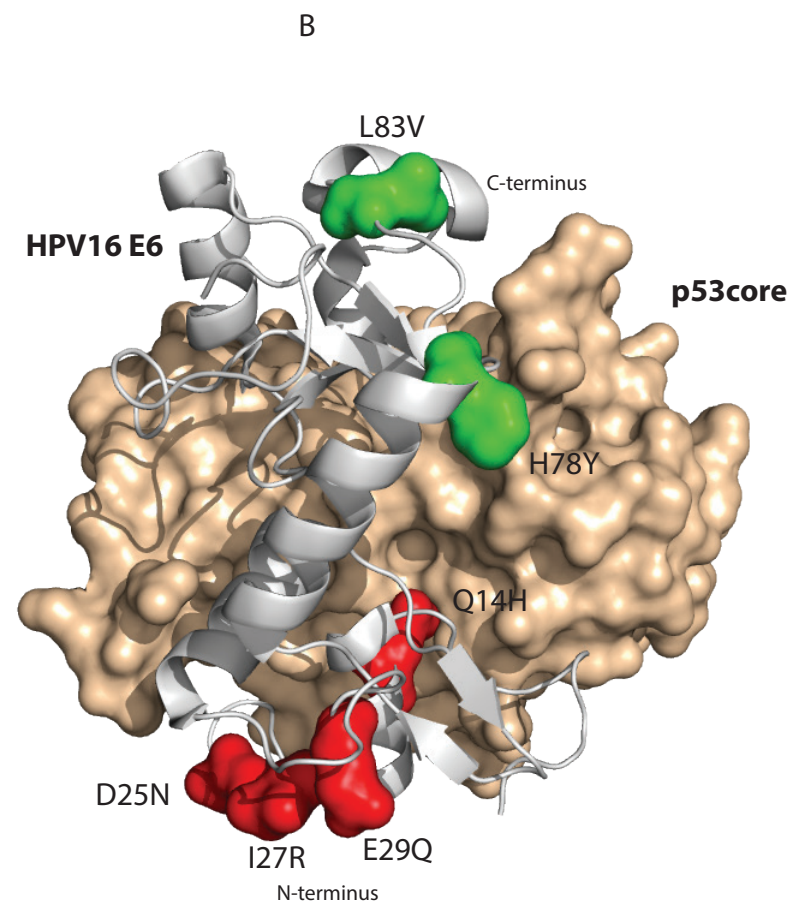

Supplement: Supplementary file 1 — Figure S1. Illustration of the non-synonymous mutations on E6 structure (colored grey) in complex with the p53core (colored wheat). Mutations Q14H, D25N, I27R and E29Q are colored red for the N-terminus mutations, while mutations H78Y and L83 V are colored green for C-terminus mutations. (A) the side view of the six non-synonymous mutations. (B) top view of the six non-synonymous mutations. PDB 4XR8 and PyMOL 2.3.0 were used to map these mutations. (PDF 2443 kb) [file 12860_2019_217_MOESM1_ESM.pdf]
